# Nebuliser Type Influences Both Patient-Derived Bioaerosol Emissions and Ventilation Parameters during Mechanical Ventilation

**DOI:** 10.3390/pharmaceutics13020199

**Published:** 2021-02-02

**Authors:** Mary Joyce, James A. McGrath, Marc Mac Giolla Eain, Andrew O’Sullivan, Miriam Byrne, Ronan MacLoughlin

**Affiliations:** 1 Aerogen Limited, Galway Business Park, H91 HE94 Galway, Ireland; mmacgiollaeain@aerogen.com (M.M.G.E.); aosullivan@aerogen.com (A.O.); rmacloughlin@aerogen.com (R.M.); 2 School of Physics & Ryan Institute’s Centre for Climate and Air Pollution Studies, National University of Ireland Galway, H91 CF50 Galway, Ireland; james.a.mcgrath@nuigalway.ie (J.A.M.); Miriam.byrne@nuigalway.ie (M.B.); 3 School of Pharmacy & Biomolecular Sciences, Royal College of Surgeons in Ireland, D02 YN77 Dublin, Ireland; 4 School of Pharmacy and Pharmaceutical Sciences, Trinity College, D02 PN40 Dublin, Ireland

**Keywords:** COVID-19, bioaerosol, vibrating mesh nebuliser, jet nebuliser, mechanical ventilation, dispersion, aerosol generating procedure, exposure, infectious disease

## Abstract

COVID-19 may lead to serious respiratory complications which may necessitate ventilatory support. There is concern surrounding potential release of patient-derived bioaerosol during nebuliser drug refill, which could impact the health of caregivers. Consequently, mesh nebulisers have been recommended by various clinical practice guidelines. Currently, there is a lack of empirical data describing the potential for release of patient-derived bioaerosol during drug refill. This study examined the release of simulated patient-derived bioaerosol, and the effect on positive end expiratory pressure during nebuliser refill during mechanical ventilation of a simulated patient. During jet nebuliser refill, the positive end expiratory pressure decreased from 4.5 to 0 cm H_2_O. No loss in pressure was noted during vibrating mesh nebuliser refill. A median particle number concentration of 710 particles cm^−3^ above ambient was detected when refilling the jet nebuliser in comparison to no increase above ambient detected when using the vibrating mesh nebuliser. The jet nebuliser with the endotracheal tube clamped resulted in 60 particles cm^−3^ above ambient levels. This study confirms that choice of nebuliser impacts both the potential for patient-derived bioaerosol release and the ability to maintain ventilator circuit pressures and validates the recommended use of mesh nebulisers during mechanical ventilation.

## 1. Introduction

Infectious diseases, caused by bacteria and viruses, can be transmitted from one person to another leading to the rapid spread of disease throughout the global population [1]. In 2019, COVID-19, resulting from infection with a novel coronavirus, Severe Acute Respiratory Syndrome Coronavirus 2 (SARS-Cov-2) was identified in Wuhan, China [2,3]. Transmission of this virus can occur from person to person in close contact with an infected person coughing, sneezing, or talking [4]. Most cases result in mild symptoms, where patient recovery is achieved without specialised treatment. However, in some instances, the disease can lead to more life-threatening illnesses such as pneumonia and acute respiratory distress syndrome with patients presenting with hypoxia and requiring supplemental oxygen [5].

Aerosol therapy is used as a means of delivering medication to critically ill patients during concurrent mechanical ventilation [6]. As such, the use of nebulisers to treat patients with COVID-19 has been widely discussed [7]. Nebulisers are the most common aerosol therapy devices used in the intensive care setting [8] and consequently, are the delivery system of choice in several ongoing COVID-19 clinical trials [9,10].

In the clinical setting, there is a risk of disease transmission to caregivers and bystanders during events known as Aerosol Generating Procedures (AGPs). These procedures include, but are not limited to, intubation, tracheostomy, manual ventilation, suctioning, bronchoscopy, non-invasive ventilation, and induction of sputum [11]. Multiple expert clinical practice guidelines were published in the last year, which focus on safety of the caregiver and mitigating the risks associated with the spread of infectious aerosol. While some guidelines suggest that nebuliser treatments are considered AGPs [12,13], there is insufficient evidence as to whether nebulisers are associated with the transmission of COVID-19 [14]. Nevertheless, guidance is provided that states that in the critically ill COVID-19 patient in receipt of ventilatory support, aerosol therapy may be used, but only with nebulisers that do not require the circuit to be broken or opened, for example, mesh nebulisers [7,15,16,17,18,19].

Inherent by design, conventional nebulisers, such as jet nebulisers (JN), typically consist of two parts: the medication cup and connection port. These must be disconnected from each other in order to refill the medication cup with drug, and in doing so, expose the inner lumen of the ventilator circuit to the external air, as well as providing an escape path for the exhaled breath or patient-derived secretions, either of which may be infectious. Conversely, vibrating mesh nebulisers (VMN) do not require the ventilator circuit to be broken for drug refill. In these nebulisers, the drug/medication cup is separated from the ventilator circuit by means of the aerosol generator component, i.e., the vibrating mesh. As such, VMN are considered closed circuit nebulisers as they can remain in-line within the pressurised ventilator circuit for periods up to 28 days, which exceeds the mean duration of ventilation in some reports of ventilator stays for COVID-19 patients [20].

Breaking of the pressurised ventilator circuit may allow for release of patient-derived bioaerosol into the local environment, which may then be unintentionally inhaled by a caregiver or bystander [21]. This route of infection can be considered the likely root cause of caregiver infection associated with ventilated patients. Countermeasures have been introduced such as clamping of the endotracheal tube (ETT) to minimise the release of particles from the patient during airway management [22], however, this is not standard clinical practice.

Beyond the risk of caregiver infection, a key clinical risk for the patient themselves that arises from this practice of breaking the ventilator circuit, is a decrease in airway pressure. Such pressure loss has the potential for lung decruitment or a reduction in airway patency [23]. Recruitment of the lung, and maintenance of optimal positive end expiratory pressure (PEEP) is considered a means of reducing the risk of ventilator-induced lung injury by keeping lung regions open that otherwise would be collapsed [24]. To date, the effect of opening the circuit on the PEEP during nebuliser drug refill has not been described and is an important consideration in the selection of nebuliser type should a reduction in PEEP be associated with some nebuliser types.

This current study investigates the release of simulated patient-derived bioaerosol into the local environment during the process of nebuliser drug refill in a simulated mechanically ventilated adult patient. This study compares two nebuliser types, open circuit JN and the closed circuit VMN, and determines whether nebuliser selection impacts upon this outcome. Further, we assess whether there are any changes in PEEP recorded at the lung during nebuliser drug refill.

## 2. Materials and Methods

### 2.1. Experimental Setup

**Figure 1:**
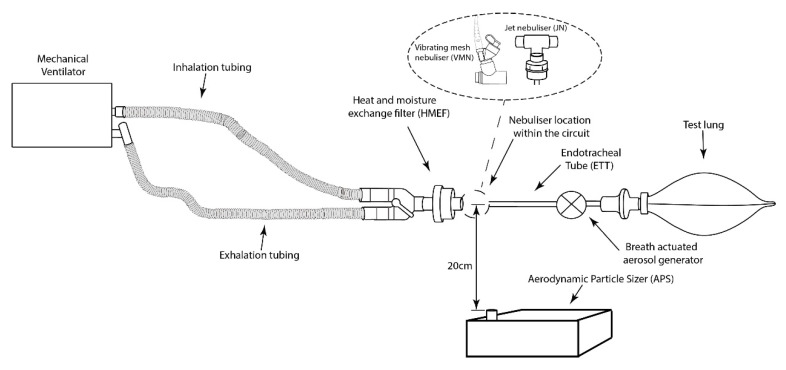
Illustration of experimental setup.

A schematic illustration of the experimental setup is presented in Figure 1. Consistent with clinical practice, a critical care mechanical ventilator (Bellavista, IMT Medical, Buchs, Switzerland) was used with a dual limb circuit (Fisher & Paykel, Auckland, New Zealand) incorporating a heat moisture exchange filter (HMEF) (Intersurgical, Wokingham, UK) at the patient side of the wye. A simulated adult ventilation pattern was used (Vt 500 mL, 15 BPM, and I:E Ratio 1:1) [25]. A VMN (Aerogen Solo, Aerogen, Galway, Ireland) or a compressed air-driven JN (Cirrus 2, Intersurgical, UK) was placed between the HMEF and the endotracheal tube (ETT) (8.0 mm, Flexicare, Mountain Ash, UK).

### 2.2. Simulated Bioaerosol

Simulated patient-derived bioaerosol exhaled by the ventilated patient was generated using a novel breath-actuated aerosol generator set up. The bioaerosol simulator was triggered to generate aerosol during the exhalation phase of the breath only, using saline (0.9%, BBraun, Sligo, Ireland) on the peak expiratory flow rate of the breath.

### 2.3. Characterisation of Bioaerosol Release to the Local Environment

Consistent with previous studies investigating fugitive medical aerosols, an Aerodynamic Particle Sizer (APS, Model 3321, TSI Inc., Shoreview, MN, USA) was used for detection of particulate number concentrations (PNC) in air [24,25]. It was placed 20 cm below the nebuliser in the circuit. Aerosol PNC were measured in the size range between 0.5 and 20 µm. Each test was five minutes in duration, with a sampling rate of five second intervals. An initial two-minute period established ambient aerosol measurements in the room, during mechanical ventilation with a closed circuit and with the bioaerosol generator activated. After two minutes, simulated addition of drug to the nebuliser was completed following the instructions provided in the respective manufacturer’s directions for use. In some clinical institutions, during procedures that require breaking of the circuit, the ETT may be clamped. To reflect this practice, during the JN testing, experiments examined scenarios where the ETT was clamped and unclamped.

**Figure 2:**
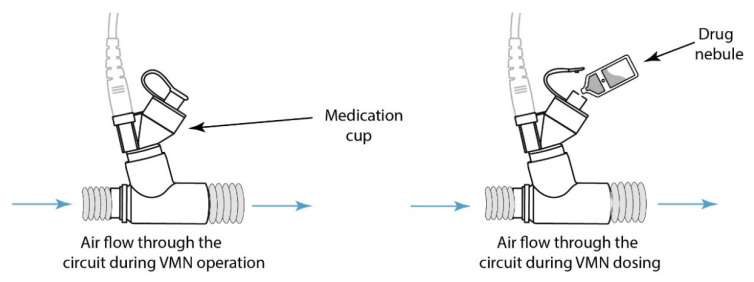
Drug refill for a vibrating mesh nebuliser (VMN) which maintains the closed ventilator circuit and mitigates the risk of release of patient-derived bioaerosol to the local environment.

**Figure 3:**
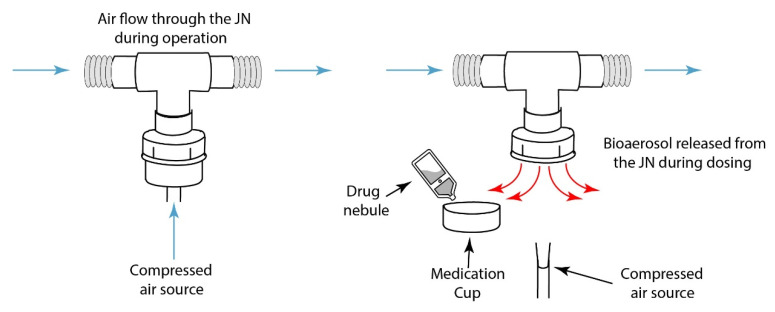
Drug refill process for a jet nebuliser (JN) which results in an open ventilator.

For the VMN, simulated refill of the nebuliser with a 2.5 mL drug dose of 1 mg/mL salbutmaol (GlaxoSmithKline Ltd., Waterford, Ireland) was completed by opening the silicon cap on the medication cup, presented in Figure 2. For the JN (with and without the ETT clamped), drug refill was completed by removing the plastic medication cup, illustrated in Figure 3. In a separate experiment for JN, the ETT was clamped by bending the ETT during drug refill. The remaining testing time monitored the presence of patient-derived bioaerosol in the local environment. All testing was completed in triplicate.

Circuit with the potential for release of patient-derived bioaerosol to the local environment.

### 2.4. Effect of Drug Refill on Ventilator Circuit Pressure

During the nebuliser drug refill process, positive end expiratory pressure (PEEP) within the ventilator circuit was measured using a pressure sensor (CITREX H5, IMT analytics, Switzerland) placed between the end of the endotracheal tube and the test lung. The pressure was continuously recorded over a 60 s period, allowing for initial pressure in the circuit (25 s), pressure during the drug refill process (10 s) and then the pressure after drug refill (25 s) to be monitored.

### 2.5. Statistical Analysis

Statistical analysis was performed using GraphPad Prism 7 (GraphPad, San Diego, CA, USA), from which aerosol concentrations were summarised using median and interquartile range (IQR). Student’s t-tests were completed between test scenarios and statistical significance was considered at *p* ≤ 0.05. All testing was completed in triplicate.

## 3. Results

### 3.1. Bioaerosol Release to the Local Environment

**Figure 4:**
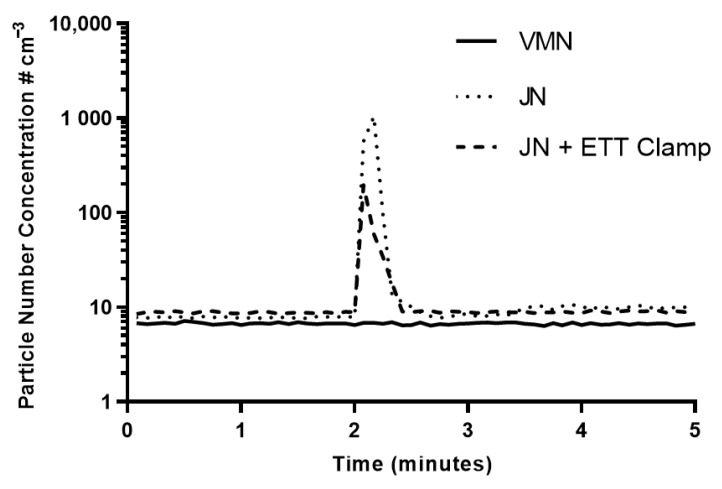
Average particulate number concentrations (PNC) for three runs for each test scenario over a five-minute period. The drug refill process was competed at the 2-minute timepoint.

Figure 4 presents the average PNC of the three runs for each test scenario. Average ambient PNC were recorded at between 6–9 particles across all tests in the initial two-minute period. For all tests, baseline ambient PNCs were subtracted to reflect the particles escaping from the circuit during simulated drug refill.

Table 1 details the median and IQR PNC across all test runs detected at the 2-minute simulated drug refill mark. For the VMN, after the simulated addition of drug to the nebuliser, there were no particle counts above ambient recorded. These results indicate that there were no particles escaping from the circuit using the VMN. In comparison, after simulated drug refill using the JN (without the ETT clamped), the median PNC was 710 cm^−3^ with an IQR of 265–1121 cm^−3^ recorded. These results indicate that there were a considerable number of particles escaping from the circuit using a JN, when compared with baseline. With the ETT clamped, the median PNC was 60 cm^−3^ with an IQR from 31–140 cm^−3^ recorded. These findings indicate that there was a significant difference in the release of patient-derived bioaerosol during drug refill using the JN in comparison to the VMN (*p* = 0.032). For the remaining period of the 5-minute test interval, an average PNC of 6–10 cm^−3^ were recorded across all tests, indicating that PNC had returned to ambient levels.

### 3.2. Ventilator Circuit Pressure

**Figure 5:**
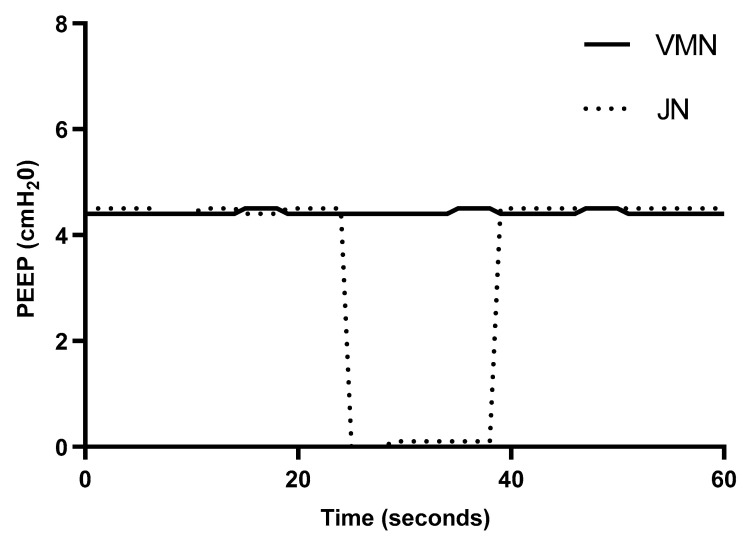
Comparison of PEEP during nebuliser drug refill of a simulated mechanically ventilated patient.

Table 2 and Figure 5 outline the average ± SD PEEP recorded during the entire one-minute test period. Using a VMN, the PEEP remained at a stable pressure of 4.4 ± 0.0 cmH_2_O for the duration of the one-minute test period, indicating that drug refill of a VMN did not affect this ventilatory parameter. In contrast, PEEP decreased from 4.5 to 0 cmH_2_O over a 10 s period during drug refill of a JN, indicating that this had a negative effect on this parameter.

## 4. Discussion

Maintaining a closed pressurised circuit during mechanical ventilation is vital in both ensuring the safe controlled ventilation of the patient, but also in mitigating the risks associated with patient-derived bioaerosol. This study demonstrates that nebuliser selection influences the release of patient-derived bioaerosol to the local environment, with a greater number of particles released into the environment during use of the open system jet nebuliser. The data confirms that patient-derived bioaerosol may be released into the local environment during mechanical ventilation when there is a break in the ventilatory circuit during refilling of jet nebulisers. During the refilling of a VMN, the ventilatory circuit remains intact, and as such, there was no release of particles during drug refill, thereby validating the guidance indicating that mesh nebulisers can be used during mechanical ventilation. Clamping the ETT during drug refill of the JN resulted in reduced level of simulated bioaerosol being released into the environment. However, this still poses a potential route of disease transmission to healthcare workers during the administration of treatment. Importantly, and for the first time, we demonstrate that refilling of a JN leads to a decrease in pressure within the circuit which in turn could lead to lung decruitment.

The intention of this study was to examine the influence of nebuliser type on both patient-derived bioaerosol emissions and mechanical ventilation parameters during nebuliser drug refill. Limitations of the study include: only a single breath type and patient cohort were considered and only a single nebuliser position in the respiratory circuit was examined. Additional research is required in order to explore these points.

There are currently no approved medications for aerosol therapy in the treatment of COVID-19. However, it is often prescribed in conjunction with mechanical ventilation to alleviate symptoms of a critically ill respiratory patient. This study highlights the importance of nebuliser selection as a deciding factor to reduce the risks associated with aerosol therapy during mechanical ventilation. While remaining cognisant of the risks associated with nebuliser selection on the transmission of infection to healthcare workers during drug administration, such as the lack of exhalation limb filtration [26,27,28,29,30], the recommended use of vibrating mesh nebulisers has now been validated.

## 5. Conclusions

This study provides key information to establish best practice in the use of nebulisers to treat a mechanically ventilated patient with an infectious disease. Here, we established that breaking the ventilator circuit for drug refill of a JN during simulated mechanical ventilation resulted in the release of patient-derived bioaerosol and a decrease in the ventilatory parameter PEEP. This current global health pandemic highlights the requirement for further studies into the transmission of infectious disease during respiratory support, and nebuliser treatment of an infected patient with respiratory complications, to establish healthcare guidelines.

## Figures and Tables

**Table 1:** Median and IQR (interquartile range) particulate number concentrations across all test runs detected for simulated drug refill at the two-minute mark.

Particulate Number Concentrations (#/cm^−3^) Median and IQR		
Vibrating Mesh Nebuliser	Jet Nebuliser	JN with Endotracheal Tube Clamped
0 (0.1–0.6)	710 (265–1211)	60 (31–140)

**Table 2:** Average ± SD PEEP (positive end expiratory pressure) during nebuliser drug refill.

PEEP (cmH_2_O) Average ± SD	
Vibrating Mesh Nebuliser	Jet Nebuliser
4.4 ± 0.0	3.5 ± 1.9

## Data Availability

The data presented in this study are available on request from the corresponding author.
